# Manual therapy followed by specific active exercises versus a placebo followed by specific active exercises on the improvement of functional disability in patients with chronic non specific low back pain: a randomized controlled trial

**DOI:** 10.1186/1471-2474-13-162

**Published:** 2012-08-28

**Authors:** Pierre Balthazard, Pierre de Goumoens, Gilles Rivier, Philippe Demeulenaere, Pierluigi Ballabeni, Olivier Dériaz

**Affiliations:** 1 Physiotherapy Department, HES-SO University of Applied Sciences Western Switzerland, HESAV, Avenue de Beaumont, Lausanne, 1011, Switzerland; 2 Centre Hospitalier Universitaire Vaudois (CHUV), Avenue Pierre Decker, Lausanne, 1011, Switzerland; 3 Clinique Romande de Réadaptation SUVACare, Avenue Grand-Champsec, Sion, 1951, Switzerland; 4 Institut de Recherche en Réadaptation et Clinique Romande de Réadaptation SUVACare, Avenue Grand-Champsec, Sion, 1951, Switzerland; 5 Institute of Social and Preventive Medicine (IUMSP), Centre Hospitalier Universitaire Vaudois (CHUV), University of Lausanne, Lausanne, Switzerland

**Keywords:** Chronic non specific low back pain, Manual therapy, Active exercises, Pain, Disability

## Abstract

**Background:**

Recent clinical recommendations still propose active exercises (AE) for CNSLBP. However, acceptance of exercises by patients may be limited by pain-related manifestations. Current evidences suggest that manual therapy (MT) induces an immediate analgesic effect through neurophysiologic mechanisms at peripheral, spinal and cortical levels. The aim of this pilot study was first, to assess whether MT has an immediate analgesic effect, and second, to compare the lasting effect on functional disability of MT plus AE to sham therapy (ST) plus AE.

**Methods:**

Forty-two CNSLBP patients without co-morbidities, randomly distributed into 2 treatment groups, received either spinal manipulation/mobilization (first intervention) plus AE (MT group; n = 22), or detuned ultrasound (first intervention) plus AE (ST group; n = 20). Eight therapeutic sessions were delivered over 4 to 8 weeks. Immediate analgesic effect was obtained by measuring pain intensity (Visual Analogue Scale) before and immediately after the first intervention of each therapeutic session. Pain intensity, disability (Oswestry Disability Index), fear-avoidance beliefs (Fear-Avoidance Beliefs Questionnaire), erector spinae and abdominal muscles endurance (Sorensen and Shirado tests) were assessed before treatment, after the 8^th^ therapeutic session, and at 3- and 6-month follow-ups.

**Results:**

Thirty-seven subjects completed the study. MT intervention induced a better immediate analgesic effect that was independent from the therapeutic session (VAS mean difference between interventions: -0.8; 95% CI: -1.2 to −0.3). Independently from time after treatment, MT + AE induced lower disability (ODI mean group difference: -7.1; 95% CI: -12.8 to −1.5) and a trend to lower pain (VAS mean group difference: -1.2; 95% CI: -2.4 to −0.30). Six months after treatment, Shirado test was better for the ST group (Shirado mean group difference: -61.6; 95% CI: -117.5 to −5.7). Insufficient evidence for group differences was found in remaining outcomes.

**Conclusions:**

This study confirmed the immediate analgesic effect of MT over ST. Followed by specific active exercises, it reduces significantly functional disability and tends to induce a larger decrease in pain intensity, compared to a control group. These results confirm the clinical relevance of MT as an appropriate treatment for CNSLBP. Its neurophysiologic mechanisms at cortical level should be investigated more thoroughly.

**Trial registration:**

Trial registration number: NCT01496144

## Background

In developed countries, 60 to 80% of the active individuals suffer from low back pain (LBP) at least once in their life [1,2]. Generally, patients with acute episode of non specific low back pain (ALBP) recover within 6 to 8 weeks, but the recurrence is frequent, and 7 to 10% of them will experience persistent pain and disabilities for more than 3 months [2-5]. Moreover, psycho-social, physical and behavioral components play an important role in the occurrence of chronic non specific low back pain (CNSLBP). Up to now, the treatment of CNSLBP is still complex and expensive and the outcome highly unpredictable [6-8].

Several CNSLBP models have been conceptualized in order to select better appropriate conservative treatments (e.g., Biomedical Model of Health; Biopsychosocial Model of Disability [8]). O’Sullivan proposed a Classification Model [9] in which peripherally or centrally mediated back pain is the driving mechanism behind the disorder, and integrated psychological factors for their potential to amplify pain and drive disability [10]. However, no strong definite clinical results and current research evidence support this perspective.

A Cortical Dysfunction Model [11] was suggested where altered brain function plays an important role in the CNSLBP. The extent of central nervous system changes may explain the duration and severity of the condition, and be responsible for over 70-80% of the variance for intensity and duration of CNSLBP. A recent study has found a correlation between cortical changes and inappropriate response to noxious stimuli, altered body perception and psychological and cognitive manifestations [11].

Clinically, the literature still recommends active exercises as an efficient conservative treatment to reduce functional disability of CNSLBP patients [12-14]. Unfortunately, this strategy may be difficult to perform due to fear that movements would induce more pain and/or injury [15]. In fact, CNSLBP patients would tend to show signs of negative anticipation, poor pain tolerance and low level of exercise/activity achievement and outcome when asked to exercise [16-20].

The impact of manual therapy on CNSLBP has been extensively investigated but results are controversial. Randomized controlled trials reported that manual therapy is more effective on physical function, mental health, physical disability and/or pain than no intervention, sham manipulation, light exercises or general active exercises [21-25]. However, an exhaustive meta-analysis involving 39 studies did not confirm the benefit of manual therapy over active exercises on long-term pain and disability [26]. Nonetheless, some studies showed that manual therapy induces an immediate analgesic effect lasting 5 to 10 minutes after manipulation [27-32]. In addition, it may interfere with the neuromuscular, autonomic and endocrine responses, produce a placebo effect and/or alter the patient’s psychological state [33].

For these reasons, the use of spinal manipulation/mobilization is favorably recommended [34,35]. Therefore, we postulate that a short-term positive effect on pain might facilitate the practice of subsequent active exercises and improve outcomes in CNSLBP. The aim of this study is first, to assess whether manual therapy has an immediate analgesic effect, and second, to compare the lasting effect on functional disability of manual therapy followed by active exercises to sham therapy followed by active exercises.

## Methods

### Subjects

The study was held at the rheumatology clinic of the Département de l’appareil locomoteur (DAL), Centre Hospitalier Universitaire Vaudois (CHUV), Lausanne, Switzerland. Patients selection was as follows: inclusion criteria: 1) aged from 20 to 65 year old, male or female, suffering from non specific low back pain with or without symptoms in the lower extremity for a period between 12 and 26 weeks; 2) the usual medication can be continued; exclusion criteria: 1) spinal fracture or surgery within the previous 6 months; 2) pregnancy; 3) neoplasia; 4) spinal infection; 5) spinal inflammatory arthritis; 6) low back pain of visceral origin; 7) severe sensitive and/or motor radicular deficit from nerve root origin of less than 6 months; 8) score of 3/5 or more on the Waddell Score [36]; 9) on sick leaves from work for 6 months or more; 10) psychiatric disorders; 11) opioid medication; 12) patient unable to collaborate (linguistic barrier; cognitive impairments); 13) radiologic abnormalities other than degenerative disease and; 14) clinical neurogenic claudication.

Before the study, we calculated the sample size needed to detect a predicted effect of an ODI score difference of 5.5 with a SD of 10 [22,37]. We predicted that 52 patients per group were needed to reach a power of 0.8 with a type I error probability of 5%. During the experimental phase, we had problems with patient recruitment and were forced, for financial reasons, to stop the recruitment process before the target sample size was reached. This decision was taken without knowledge of study findings.

All eligible patients were given written information about the study and were asked to provide written consent before participating. Afterwards, they were asked to attend an initial evaluation visit in order to perform clinical tests and fill in self-questionnaires, supervised by a physiotherapist not involved in the patients’ therapies and blinded to the treatment groups. The same physiotherapist supervised subsequent patients’ evaluation visit after the 8th therapeutic session, and at 3 and 6 months after the end of the treatment. The study protocol was approved by the Ethics Committee of Clinical Research, Faculty of Biology and Medicine, University of Lausanne, Switzerland.

### Randomization

Following the initial evaluation visit, patients were randomly assigned to their treatment group. Concealment allocation was performed by using a randomized table of numbers [38], from which every four consecutive numbers were retained. Individual index cards with the corresponding number were folded and placed in consecutively numbered, sealed opaque envelopes. Even numbers were allocated to the manual therapy (MT) group and odd numbers to the sham therapy (ST) group.

### Treatments

Treatments consisted of a physiotherapy evaluation and 8 therapeutic sessions (1–2 sessions per week) over a period of 4 to 8 weeks.

The *physiotherapy evaluation* (45 minutes) included: 1) a standard physiotherapy assessment for non specific low back pain [39]; 2) an educational information on the low back anatomy and biomechanics, ways to protect the spine during activities of daily living and rest during episodes of pain (presented in a 6-page booklet); 3) 2 home mobility exercises (pelvic tilt and low back lateral flexion, in supine), to be performed daily, twice a day, 2 sets of 10 repetitions. After the 3^rd^ or 4^th^ therapeutic session, the recommendation of home exercises changes to stretching and motor control exercises (see active exercises for dosage and progression). Home exercises were reviewed at the beginning of each therapeutic session and recorded daily by patients in a diary.

The *therapeutic sessions* (30 minutes) consisted of: for the MT group, 5 to 10 minutes of MT intervention followed by active exercises (AE); for the ST group, 5 to 10 minutes of ST intervention followed by AE.

#### I. MT/ST intervention

The MT intervention, performed by a single physiotherapist of 15 years of experience, comprises the use of one (or more) of the following techniques:

• Passive accessory intervertebral movements, a central or unilateral postero-anterior pressure applied on painful or stiffed vertebral segment(s) with the patient lying prone [39].

• Muscle-energy techniques, a hold-relaxed technique performed on an ilium dysfunction with the patient side lying [25].

• High velocity, low amplitude dynamic thrust (manipulation), a rotational-lateral flexion thrust performed on a stiffed vertebral segment(s) with the patient side lying [23,40].

The ST intervention, delivered by 2 physiotherapists of 5 and 25 years of working experience at the rheumatology unit of the DAL (CHUV), relied on detuned ultrasound on the patient’s painful and/or inflammatory site. The patient did not know the ultrasound was inactivated and, therefore, ineffective. The choice of the therapist depended exclusively on immediate availability, work schedule and vacation.

#### II. Active exercises (AE)

Before the start of the clinical phase, the 3 treating therapists agreed on a protocol of therapeutic exercises (type; dosage; progression).

• Mobility exercises throughout the 8 therapeutic sessions to improve patient’s spinal range of motion and pain. For the first 2 sessions, pelvic tilt and low back lateral flexion exercises were performed in the supine position, 3 sets of 5 to 10 repetitions. From session 3, the same exercises were adapted in sitting on a stable plane, then on a swiss ball.

• Passive stretching exercises after the 2nd session, for muscle groups that tend to shorten (erector spinae, hamstring, iliopsoas, rectus femoris, piriformis), to relieve muscular tension and improve low back mobility. They were performed 3 times for 20 seconds.

• Motor control exercises at the 4th session for active recruitment of stabilizing trunk muscles [41]. Patients were asked to contract their transverse abdominus and/or multifidus muscle at 20% of maximum voluntary contraction, under visual and tactile supervision, for 10 to 30 seconds, 5 to 10 times. At first, the exercises were performed supine, then seated and, finally, in the standing position. Progression went from static to dynamic contraction.

• Strengthening exercises at the 6th or 7th session to increase strength of weak superficial trunk muscles. They were performed at 60–70% of maximum voluntary contraction, against the resistance of an adapted rubber band, 2 sets of 20 repetitions.

The same physiotherapist who performed the preceding MT/ST intervention supervised the active exercises.

At the end of the 8 therapeutic sessions, no particular recommendations were given to patients but to continue their exercises if desired. This issue was not investigated at the 3- and 6-month evaluation visits.

### Outcome measures

Immediate analgesic effect, evaluating the MT/ST intervention’s efficiency, was obtained by measuring pain intensity (Visual Analogue Scale – *VAS-pain*, immediate effect) before and immediately after the manual therapy or detuned ultrasound intervention at each therapeutic session. To evaluate the treatment efficiency, pain intensity (*VAS-pain*, average 48-hour pain), disability (Oswestry Disability Index - *ODI*), fear-avoidance beliefs (Fear-Avoidance Beliefs Questionnaire - *FABQ*) and Sorensen and Shirado tests were determined before treatment, after the 8^th^ therapeutic session, and at 3- and 6-month after the end of treatment.

### Primary outcomes

1) *VAS-pain* is a self-report of clinical pain intensity, consisting of a 10 cm horizontal line scale on which is added the statements “no pain” on the left and “maximum intensity of pain” on the right [42]. Firstly, to evaluate *VAS-pain* (immediate effect), patients were asked to rate their *current pain* twice at each therapeutic session. Secondly, to evaluate *VAS-pain* (average 48-hour pain), patients were asked to rate *their average pain during the last 48 hours* before each evaluation visit. VAS-pain ratings are reported to have good reliability and concurrent validity when compared to other methods of pain measurement [43]. 2) *ODI* is a self-rating questionnaire used to evaluate functional physical disability [44]. It includes 10 sections of 6 propositions, each of them rated on a 0–5 scale; the maximum possible score is 50. Relative values are reported (total score/total possible score × 100%). Higher is the score, worst is the disability. For CLBP, it has good level of internal consistency and test-retest reliability [45].

### Secondary outcomes

1) *FABQ* measures level of fear and avoidance beliefs about work and physical activity in patients with low back pain [46]. The instrument consists of two subscales, a four-item physical activity subscale (FABQ-pa), and a seven-item work subscale (FABQ-wk). Each item is scored from 0 to 6 and summed to produce the subscale score. Possible scores range from 0–28 and 0–42 for the FABQ-pa and FABQ-wk, respectively. This questionnaire has good level of internal consistency and test-retest reliability [42,47]. 2) *Sorensen test* evaluates the erector spinae muscles endurance [48]. 3) *Shirado test* assesses the abdominal muscles endurance [49]. Sorensen and Shirado tests are relatively safe, easy to perform and have high reliability in subjects with and without non specific low back pain.

### Statistical analysis

The effect of intervention (MT vs. ST), time and the intervention-time interaction on the immediate effect of the intervention (*VAS-pain*, immediate effect) at each time point (pain after minus pain before) was analysed by means of random coefficient linear mixed models. In these models, the effects of the independent variables are allowed to vary between subjects. In other words, subjects were allowed to have their individual slope for the outcome over time. To control for potential bias due to regression to the mean, pain measured before each therapeutic session was entered in the model as time-varying covariate.

The effect of treatment (MT + AE vs. ST + AE), time and the treatment-time interaction on the six outcome variables pain intensity (*VAS-pain*, average 48-hour pain), ODI, FABQ-wk, FABQ-pa, Sorensen and Shirado, evaluated after the 8^th^ therapeutic session, and at 3 and 6 months after the end of treatment, were estimated by means of random coefficient linear mixed models. The outcomes’ baseline values (measures before treatment) were entered as a covariate to adjust for baseline differences between treatments. First, the analysis was performed with the interaction. When the effect was not significant, the analysis was repeated without the interaction.

Because two primary outcomes were assessed (i.e., functional physical disability and pain), we considered an alpha level of 0.025 for those two outcomes. The conventional alpha = 0.05 was kept for inference about the secondary outcomes.

## Results

**Figure 1:**
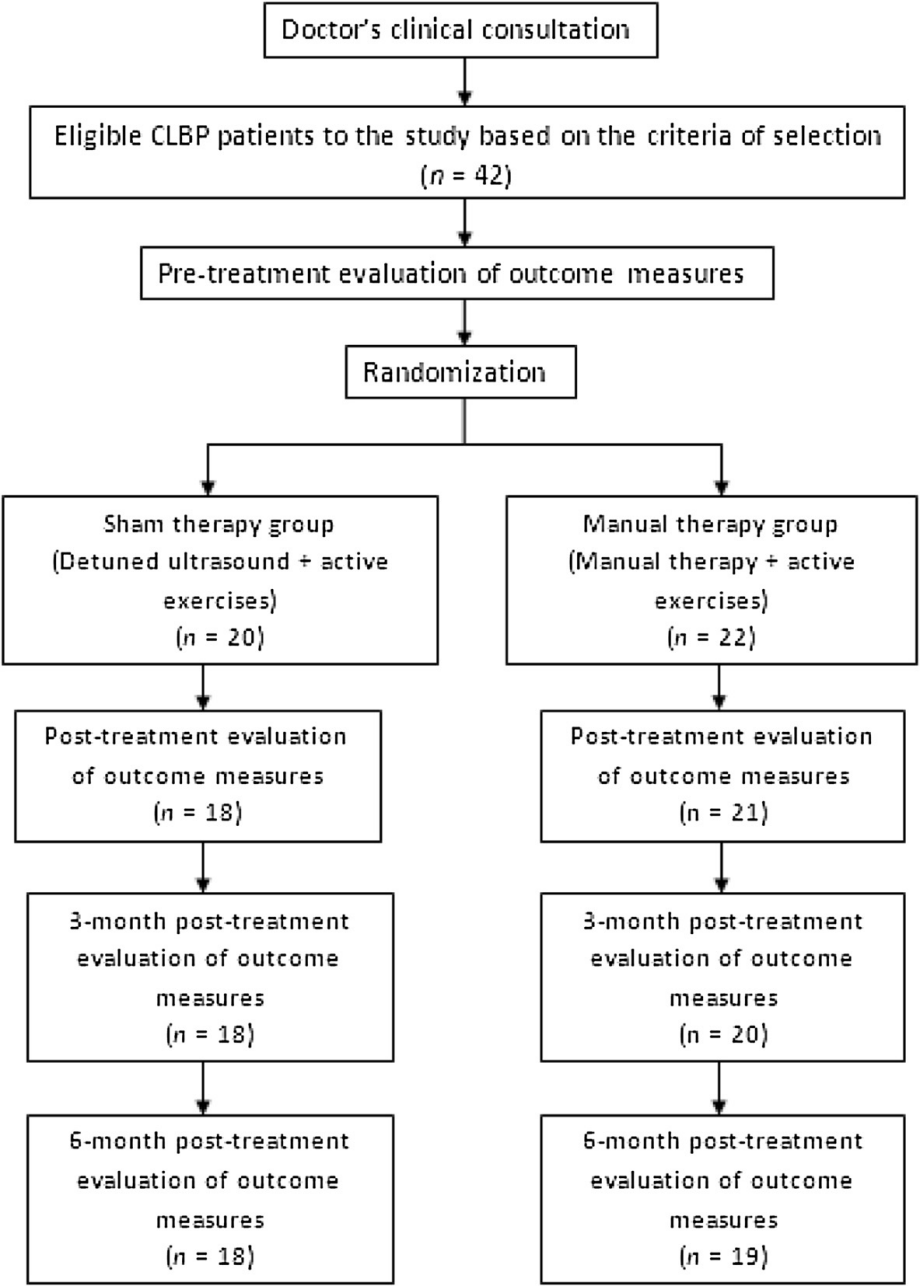
Flow chart demonstrating patient recruitment, study design and timing of data collection of Treatment groups.

A total of 42 subjects were eligible for the study: 22 in the MT group, 20 in the ST group. The MT group had one patient stopping his treatment after the sixth session because of severe pain, another being unreachable for the 3-month post-treatment evaluation, and a third patient becoming unreachable for the 6-month post-treatment evaluation. For the ST group, two patients did not attend subsequent evaluations after the end of their therapeutic sessions. One had intense pain and rest was recommended by the doctor, and the other cancelled his appointment and became unreachable (Figure 1). The dropped-out rate of patients varies from 10 and 14%, ST and MT groups respectively (Tables 1 and 2).

**Table 1:** Baseline characteristics of subjects (n = 42)

**Variable**	**Sham therapy + Active exercises**	**Manual therapy + Active exercises**
	**(n = 20)**	**(n = 22)**
Age (yrs)	42 ± 12	44 ± 12
Gender, ♀ (#, (%))	6 (30)	8 (36)
Body Weight (kg)	76 ± 13	71 ± 13
Body Height (cm)	174 ± 8	171 ± 9
Body Mass Index (kg/m^2^)	25 ± 5	24 ± 3
On Sick leaves (#, (%))	3 (15)	4 (18)
Smokers (#, (%))	4 (20)	6 (27)

All values are means ± SD, except for gender, on sick leaves and smokers.

**Table 2:** Values of self-questionnaires (VAS-pain (average 48-hour pain); ODI; FABQ-wk; FABQ-pa) and clinical tests (Sorensen; Shirado) for the MT and ST groups before treatment, after treatment, and at 3 and 6 months after the end of treatment

**Variable**	**Time**	**ST group**		**MT group**	
		**n**	**Mean ± SD**	**n**	**Mean ± SD**
VAS-pain Average 24 hour (mm)	Pre	22	65 ± 22	20	53 ± 20
	Post	21	41 ± 29	18	28 ± 21
	3 months	20	42 ± 32	18	18 ± 17
	6 months	19	38 ± 32	18	23 ± 17
ODI	Pre	22	32 ± 14	20	30 ± 13
	Post	21	26 ± 15	18	20 ± 15
	3 months	20	26 ± 21	18	16 ± 14
	6 months	19	26 ± 25	18	16 ± 11
FABQ-wk	Pre	22	20 ± 14	20	21 ± 11
	Post	21	17 ± 13	18	17 ± 14
	3 months	20	21 ± 13	18	18 ± 15
	6 months	19	19 ± 13	18	18 ± 14
FABQ-pa	Pre	22	15 ± 7	20	11 ± 8
	Post	21	15 ± 7	18	13 ± 8
	3 months	20	15 ± 8	18	11 ± 7
	6 months	19	13 ± 8	18	11 ± 9
Sorensen (sec.)	Pre	22	53 ± 50	20	57 ± 51
	Post	21	67 ± 49	18	68 ± 58
	3 months	20	78 ± 46	18	57 ± 50
	6 months	19	86 ± 61	18	76 ± 47
Shirado (sec.)	Pre	22	98 ± 87	20	96 ± 107
	Post	21	102 ± 84	18	128 ± 112
	3 months	20	144 ± 107	18	116 ± 99
	6 months	19	158 ± 140	18	114 ± 73

VAS-pain, Visual Analogue Scale of pain; ODI, Oswestry Disability Index; FABQ-wk, Fear-Avoidance Beliefs Questionnaire Work Subscale; FABQ-pa, Fear-Avoidance Beliefs Questionnaire Physical Activity Subscale; SD, standard deviation.

MT group = Manual therapy group; ST group = Sham therapy group.

Pre = before treatment; Post = after treatment; 3 and 6 months = 3 and 6 months after the end of treatment; n = number of patients.

For all scales and questionnaires, the score increases with severity of pain or symptom, except for the Sorensen and Shirado tests, wich act conversely.

**Figure 2:**
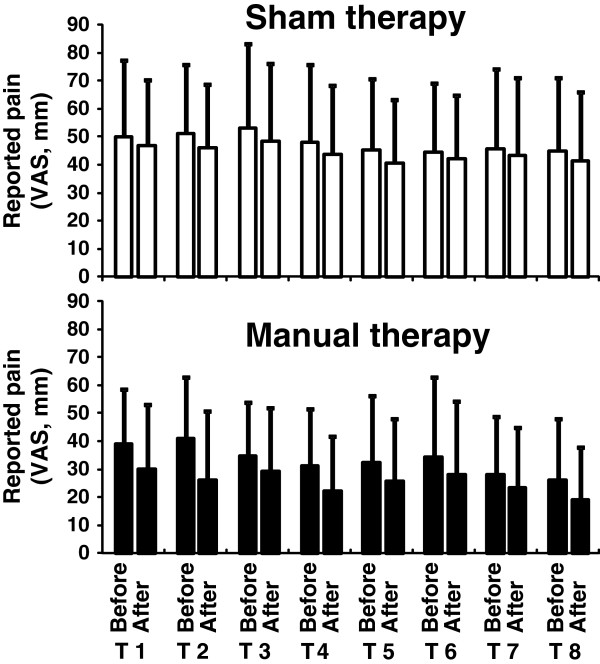
**Effect of sham therapy (ST) and manual therapy (MT) on VAS-pain before-immediately after the intervention, for the therapeutic sessions 1 to 8.** Manual therapy induces a greater immediate effect (i.e., difference between after and before) than sham therapy.

For MT/ST intervention, the immediate effect of intervention was in favor of manual therapy over detuned ultrasound. Independently from the therapeutic session and each session’s baseline measure, MT intervention showed a greater decrease in mean pain level compared to ST intervention (mean difference between interventions: -0.76 VAS units; 95% CI: -1.22 to −0.30) (Table 3; Figure 2).

**Table 3:** Results of mixed models for primary outcomes

**Outcome**	**Predictors**	**Coeff**	**95% CI**	***P***
Immediate effect on pain (base line = before each session)				
VAS-pain Immediate effect	Intervention	−0.76	[−1.22;-0.30]	0.001*
	Time	0.02	[−0.03;0.06]	0.484
	Pain baseline	−0.27	[−0.33;-0.20]	<0.001
	Constant	0.81	[0.29;1.33]	0.002
Treatment effect on time (base line = before treatment)				
VAS-pain Average 24 hour	Treatment	−1.24	[−2.37;-0.11]	0.032
	Time	−0.20	[−0.69;0.29]	0.426
	Pain baseline	0.43	[0.17;0.69]	0.001
	Constant	1.68	[−0.37;3.73]	0.108
ODI	Treatment	−7.14	[−12.80;-1.52]	0.013*
	Time	−1.80	[−3.92;0.33]	0.097
	ODI baseline	1.95	[1.50;2.39]	<0.001
	Constant	0.40	[−7.88;8.68]	0.924

* Statistical significance for primary outcomes (*P <* 0.025).

Since the time by treatment interactions were not statistically significant, the analyses were repeated after dropping the interaction terms.

Coeff = regression coefficients: same interpretation as for ordinary (least square) regression.

CI = confidence intervals; VAS-pain, Visual Analogue Scale of pain; ODI, Oswestry Disability Index.

VAS-pain (immediate effect) was the pain difference before and after each session, and time was the number of the 8 therapeutic sessions.

VAS-pain (average 24 hour) and ODI were calculated over time, i.e., before (baseline) and after the 8^th^ therapeutic session, as well as at 3 and 6 months after the end of the treatment.

**Figure 3:**
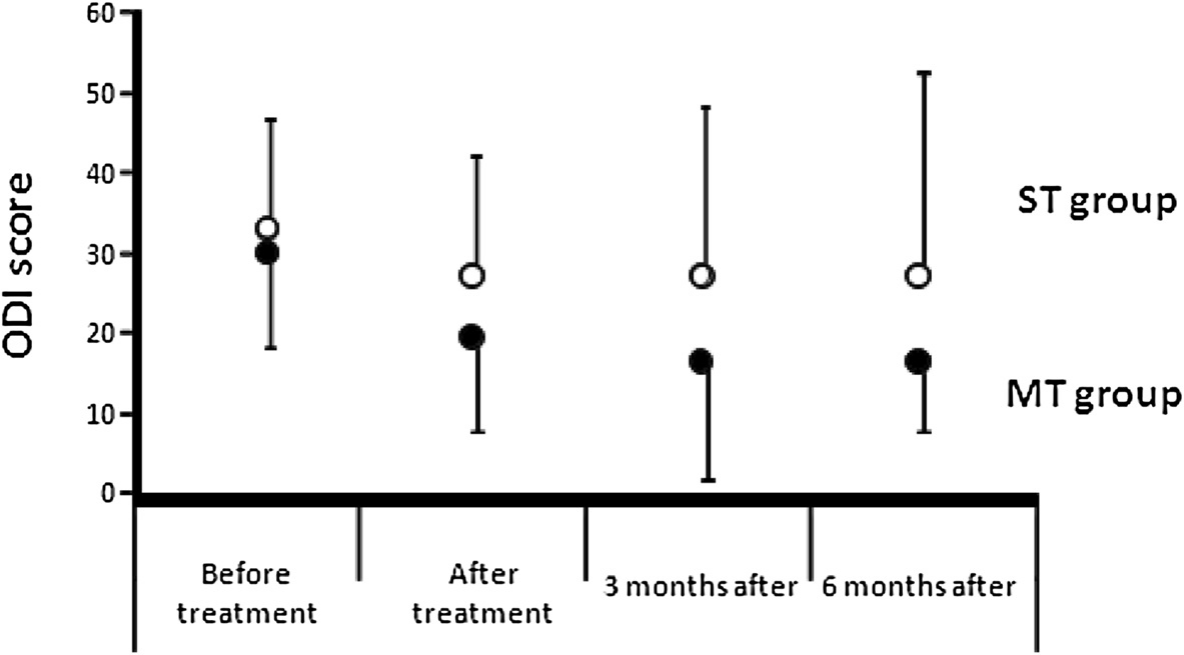
Evolution of functional disability (ODI score) for the Manual Therapy and the Sham Therapy groups over time.

For MT + AE/ST + AE treatment, independently from the time after treatment and from baseline measurement, a trend to a larger decrease in pain intensity (average 48-hour pain) and a statistically significantly reduced disability were observed in favor of the MT group over the ST group (VAS-pain mean group difference: -1.24; 95% CI: -2.37 to −0.30; *P =* 0.032, statistically not significant at the 0.025 level. ODI mean group difference: -7.14; 95% CI: -12.8 to −1.52; *P =* 0.013) (Table 3; Figure 3).

The effect of MT + AE/ST + AE treatment on the Shirado score was dependent on the time of measurement (statistically significant treatment by time interaction, Table 4). Following effects were derived from the model on Table 4. Just after treatment (time = 1), there is insufficient evidence that mean Shirado score differs between treatment groups (mean group difference: 17.71; 95% CI: -19.67 to 55.00; *P =* 0.352). Three months after treatment (time = 2), there was still insufficient evidence for a treatment effect but the MT group has a lower mean compared to the ST group (mean group difference: -21.94; 95% CI: -9.04 to 15.13; *P =* 0.246). Six months after treatment (time = 3), the mean Shirado score had dropped further in the MT group compared to the ST group; the group mean differences at this time point are statistically significant (mean group difference: -61.59; 95% CI: -117.45 to −5.74; *P =* 0.031).

**Table 4:** Results of mixed models for secondary outcomes

**Outcome**	**Predictors**	**Coeff**	**95% CI**	***P***
FABQ-wk	Treatment	−1.95	[−5.12;1.22]	0.228
	Time	1.10	[−0.25;2.45]	0.111
	FABQ-wk baseline	0.90	[0.78;1.03]	<0.001
	Constant	−0.75	[−5.00;3.50]	0.729
FABQ-pa	Treatment	0.02	[−3.33;3.36]	0.992
	Time	−0.64	[−1.68;0.39]	0.223
	FABQ-pa baseline	0.61	[0.38;0.83]	<0.001
	Constant	6.55	[2.01;11.10]	0.005
Sorensen	Treatment	−9.69	[−31.70;12.30]	0.388
	Time	6.64	[−1.63;14.90]	0.116
	Sorensen baseline	0.51	[0.29;0.73]	<0.001
	Constant	33.19	[7.88;58.50]	0.010
Shirado	Treatment	57.40	[1.13;113.60]	0.046*
	Time	29.00	[7.42;50.50]	0.008
	Treatment by time	−39.70	[−69.00;-10.00]	0.009
	Shirado baseline	0.72	[0.54;0.90]	<0.001
	Constant	8.36	[−36.3;53.10]	0.714

* Statistical significance for secondary outcomes (*P <* 0.05).

Since the treatment by time interactions were not statistically significant for FABQ-wk, FABQ-pa and Sorensen, the analyses for these outcomes were repeated after dropping the interaction terms.

Coeff = regression coefficients: same interpretation as for ordinary (least square) regression.

CI = confidence intervals, FABQ-wk, Fear-Avoidance Beliefs Questionnaire Work Subscale; FABQ-pa, Fear-Avoidance Beliefs Questionnaire Physical Activity Subscale; Sorensen and Shirado tests evaluate the erector spinae and abdominal muscles endurance, respectively.

All variables were calculated over time, i.e., before (baseline) and after the 8 treatments as well as at 3 and 6 months after the end of the treatment.

This study provides insufficient evidence for an effect of MT + AE on FABQ-wk, FABQ-pa or Sorensen scores (Table 4).

## Discussion

To the authors’ knowledge, this is the first controlled study to assess the efficacy of spinal manipulation/mobilization followed by specific active exercises. The main original result of this study is that manual therapy, immediately followed by active exercise, accelerates reduced disability in CNSLBP patients.

Several studies, with various designs, tried to assess the effect of manual therapy on CNSLBP, e.g., manual therapy alone [4,41] or with exercises [23,25] compared to exercises alone [23,41] or other interventions (physician consultation with patient education; motor control exercises with cognitive-behavioral therapy; group exercises with cognitive-behavioral therapy) [4,25]. None of these studies included a ST intervention (placebo). Therefore, the authors believe the design of the present study, with a true placebo intervention (i.e., the patient does not know that the ultrasound is ineffective), may allow to isolate the real effect of manual therapy.

Only one controlled study investigated several interventions, like for instance the effect of manual therapy (vs. placebo) and specific active exercises, with a comparable design [50]. In their study, the MT intervention involved primarily muscle energy technique, and the ST intervention consisted of placing patients in a controlled position that would potentially correct their musculoskeletal dysfunction, without having the muscle energy performed. For the group “manual therapy followed by specific active exercises”, at the end of the 6 therapeutic sessions, they observed a significant decrease in pain intensity, but not in disability, measured by the Quebec Back Pain Disability Scale (QBPDS). No long term results were collected by the authors.

Besides, this study confirms the immediate analgesic effect of manipulation/mobilization already reported in the literature [27,28,31]. No other studies assessed, throughout 8 consecutive therapeutic sessions, the immediate analgesic effect of manual therapy, and compared it to a “real” placebo treatment. Hemmilä et al. [22] assessed the effect of bone setting (described by the authors as gentle mobilization of the sacroiliac joint and the spinal vertebrae) on back pain to: i) light exercises and; ii) physiotherapy (massage with specific mobilizations and manual traction) during 10 one-hour therapeutic sessions over a 6-week therapy period. No significant differences were recorded between therapies at 12 months after randomization, although more patients in the bone-setting group notified their pain remained “improved”. Ferreira et al. [4] compared the effect of spinal mobilization on pain to: i) motor control exercises with cognitive-behavioral therapy and; ii) group exercises with cognitive-behavioral therapy. It showed no significant decrease at 8 weeks, 6 and 12 months compared to baseline. For both studies, no data on pain intensity were taken before and immediately after each therapeutic session, i.e., no analysis was performed to evaluate the immediate effect of manipulation/mobilization over the other therapies.

In this study, the VAS-pain scores present lower values under the MT group throughout time (i.e., before and after the 8 therapeutic sessions). However, a comparison of pain reduction between treatments (MT + AE vs. ST + AE) shows a significant difference in slopes in favor of MT group when the interaction is dropped (Table 3). This strongly suggests that the analgesic effect of MT combined with exercises can be efficient to decrease pain for CNSLBP. The reported analgesic effect of manual therapy (i.e., the immediate effect) may allow the patient to perform better/more accurate active exercises. Unfortunately, no measure of patients’ activity level was performed during and between therapeutic sessions, or after the end of the treatment. Also, it has been suggested that manual therapy may have a facilitator effect on muscle activation [33,51]. It may also help patients to perform more accurate exercises. Both hypotheses may be supported by putative neurophysiologic mechanisms induced by manual therapy at peripheral, spinal cord and supraspinal levels [33].

These results in functional disability are quite similar to those of other studies [22,23,25]. Only Ferreira et al. [4] did not observe greater improvement in disability and pain for their MT group. Again, as mentioned above, the relevance of comparing groups offering fundamentally different interventions does not allow isolating the effect of manual therapy.

In the present study, the results on the FABQ questionnaire are interesting. No significant improvement in the FABQ-pa was observed (Table 4). This result appears to contradict earlier results, i.e., a good correlation between the FABQ-pa and disability (or rehabilitation outcome) [52,53], but is in accordance with the weak association between perceived disability and fear-avoidance belief reported by Waddell et al. [46] and Sieben et al. [54]. As for the FABQ-wk, the present result is in agreement with the literature (Table 4), i.e., a low correlation between the FABQ-wk and disability [55].

There is not sufficient evidence that MT + AE has a positive influence on the static abdominal endurance, i.e. Shirado test (see Results). For unknown reasons, the patients treated with MT + AE presented a more pronounced drop of abdominal endurance than ST + AE when the treatment sessions were stopped. We cannot explain these results and further studies are clearly needed to better understand the relationship between MT, AE and abdominal endurance.

This study presents some limitations. For instance, patient recruitment was provided exclusively by the rheumatology clinic of a University hospital, which does not reflect the whole spectrum of the CNSLBP population. This may have contributed in selecting patients with more inflammatory signs and higher severity of disease. However, strict criteria of selection (e.g., CNSLBP and sick leaves from work of less than 6 months) were used in order to exclude patients with bad prognosis, e.g., severe disabilities and long-term unemployment [56]. For these reasons, the present results cannot be generalized to the CNSLBP population.

Besides, the large standard deviations obtained for the self-questionnaires and clinical tests values indicate the patients were heterogeneous. In addition, the population was smaller than the one estimated prior to study which produces statistical power problems. However, the number of subjects per group was comparable to the studies of Aure et al. [23], Rasmussen-Barr et al. [41] and Geisser et al. [50]. Despite these limitations, the authors were able to observe that, with this protocol design, manual therapy followed by active exercises was efficient on various patients with CNSLBP. Nonetheless, further studies with a larger number of patients are obviously needed in order to assess the exact role of fear avoidance in this therapy.

All data available from patients, even if they dropped out of the study, were integrated in the study analysis. Since the dropped out rate was low and similar in both treatments, no special statistical analyses were performed.

## Conclusions

The present study confirms the immediate analgesic effect of manual therapy for CNSLBP. Followed by specific active exercises, it reduces significantly functional disability and tends to induce a larger decrease in pain intensity, compared to a control group. However, these results of our pilot study need to be confirmed by future studies with appropriate sample sizes.

Recent evidence tend to demonstrate that CNSLBP is largely characterized by structural, functional and neurochemical cortical modifications [11,57]. In the near future, improving the knowledge of the precise neurophysiologic mechanisms of manual therapy at cortical level seems essential in order to validate the choice of this therapy for CNSLBP.

## Competing interests

The authors declare that they have no competing interests.

## Authors’ contributions

PB participated in the conception, design and coordination of the study, contributed to interpretation of data, and helped to draft the manuscript. GR have been involved in drafting the manuscript or revising it critically for important intellectual content. PD carried out the evaluation of patients and contributed in acquisition of data. PiBall performed the statistical analyses and contributed to the manuscript. PG participated in the initial selection of patients and revisiting the manuscript for important intellectual content. OD participated in the design of the study, helped in the interpretation of data and drafting the manuscript. All authors read and approved the final manuscript.

## Pre-publication history

The pre-publication history for this paper can be accessed here:

http://www.biomedcentral.com/1471-2474/13/162/prepub
